# Frost Durability of Self-Compacting Concrete Prepared with Aeolian Sand and Recycled Coarse Aggregate

**DOI:** 10.3390/ma16196393

**Published:** 2023-09-25

**Authors:** Hao Yan, Qing Liu, Fengxia Han, Shan Liu, Tong Han, Bowen He

**Affiliations:** 1 College of Civil Engineering and Architecture, Xinjiang University, Urumqi 830017, China; 107552104209@stu.xju.edu.cn (H.Y.); hant321@xju.edu.cn (T.H.); 107552104147@stu.xju.edu.cn (B.H.); 2 Key Laboratory of Building Structure and Seismic Resistance of Xinjiang, Urumqi 830017, China; fxhan@xju.edu.cn (F.H.); cainhide@163.com (S.L.)

**Keywords:** aeolian sand, recycled coarse aggregate, self-compacting concrete, freeze–thaw cycles, damage model

## Abstract

Aeolian sand (AS) and recycled coarse aggregate (RCA) can be reasonably utilized as green materials for concrete modification. The paucity of natural sand and gravel in the construction industry is anticipated to be remedied by the use of these two eco-friendly concrete ingredients. This is incredibly important for environmental protection. Study on the damage law of self-compacting concrete with the addition of AS and RCA (ARSCC) under severely cold conditions is of great significance for the promotion and implementation of this material. In this study, 12 groups of ARSCC specimens were prepared for freeze–thaw cycle experiments, with AS substitution rates of 0, 20%, 40%, and 60% as well as RCA replacement rates of 0, 25%, and 50%. Then, the degradation mechanism of ARSCC freeze–thaw damage was discussed from both macroscopic and microscopic perspectives via mass loss rate (W_n_), relative dynamic modulus of elasticity (P_n_), bubble spacing factor, and SEM analysis. Finally, the response surface method was utilized to determine the damage variable. A freeze–thaw damage model for ARSCC was developed based on the Weibull distribution and Grey theories. The results showed that the P_n_ could reflect the evolution law of the internal structure of ARSCC. Appropriate addition of AS to fill the large, harmful pores in RCA would inhibit freeze–thaw damage of ARSCC. The optimum substitution rates of AS and RCA were determined to be 20–40% and 25–50%, respectively. In addition, the values obtained from theoretical damage modeling and experiments were in good agreement. The acquired damage model had the potential to predict ARSCC damage under freeze–thaw cycles.

## 1. Introduction

Nowadays, there is an increasingly high demand for environmental protection and sustainable development in the construction industry [1,2,3]. Recycled aggregate concrete (RAC) is an eco-friendly and sustainable material that plays a significant role in promoting the development of green architecture and mitigating the adverse environmental effects of construction waste [4]. On the basis of material source, coarse aggregates are categorized as recycled and natural. The variability in the number of pores in RCA and surface old mortar leads to significant differences between RCA and natural coarse aggregate (NCA) regarding the high water absorption and low strength [5]. These differences result in the lower durability of RAC compared to natural aggregate concrete (NAC), which is mainly attributed to the quality and content of RCA [6]. To alleviate the effect of RCA on the durability of RAC, AS can be introduced into RAC [7]. On the one hand, the appropriate addition of AS forms a continuous gradation with river sand as well as filling the large and harmful pores in the RCA. On the other hand, volcanic ash reactions in the active components of AS produce C–S–H gel, which can fill dangerous pores and enhance the internal compactness of concrete, thus improving the durability of concrete [8,9]. The major chemical composition of AS is SiO_2_, which is characterized by uniformly tiny particles, homogeneous composition, abundant reserves, and easy availability. Desertification has seriously affected several countries around the world, including China. In particular, the western region of China has experienced severe AS-induced desertification, where Xinjiang alone accounts for approximately 60% of the national desert area [10]. If AS in Xinjiang could be applied to concrete, this could significantly alleviate the shortage of construction materials in this region and promote the harmonious coexistence of humans and nature. Therefore, the usage of AS in RAC instead of natural river sand to produce ARSCC could potentially reduce the reliance of buildings on natural river sand and gravel, conserve resources, as well as ease ecological pressure.

Compared with ordinary concrete, the greater water absorption of AS and the higher porosity of RCA make ARSCC more susceptible to freeze–thaw damage, so the frost resistance of ARSCC is a critical indicator used to evaluate its durability [11,12,13]. Currently, the representative theories that elucidate the law and mechanism of concrete freeze–thaw damage are Powers’ theory of hydrostatic and osmotic pressures [14,15] and Setzer’s micro-ice lens theory [16]. Many scholars have concluded that contents of AS and RCA significantly affect the frost resistance of concrete [17,18,19,20,21,22]. Huda et al. [23] revealed experimentally that the frost resistance of recycled concrete tended to decrease with increasing RCA. Gokce et al. [24] found that microcracks were first concentrated in the old mortar attached to RCA. Freeze–thaw cycles accelerated the development of cracks, which eventually penetrated the fresh mortar and caused damage. Bai et al. [25] analyzed the main microscopic pore parameters affecting the frost resistance of wind-calcified sand concrete. They suggested that the workability and mechanical properties of wind-calcified sand concrete increased and then decreased as the AS substitution rate increased and that the optimum substitution rate was 30%. Wei et al. [26] discussed the damage caused by freeze–thaw cycles on AS lightweight aggregate concrete from both micro- and macroscopic points of view. They confirmed that the optimum substitution rate of AS was 20–30% of river sand. There have been relevant studies on the fundamental properties and frost resistance of AS concrete and recycled concrete [27,28,29,30,31,32,33,34]. However, there are limited reports on the freeze resistance of AS utilized in RAC. Therefore, it is imperative to study the damage mechanism of ARSCC during freeze–thaw cycles.

Over the past decades, numerous studies have been conducted on the damage model of concrete affected by freeze–thaw cycles. Bu et al. [35] and Chen et al. [36] evaluated the service life of concrete under different conditions from different perspectives using the Weibull distribution model. Xiao et al. [37] used the damage theory to develop a damage prediction model for concrete under the action of freeze–thaw cycles. Gong et al. [38] investigated the various factors affecting the mechanical characteristics of iron sand-modified cement mortar based on the Grey theory. Also, classic mathematical formulas of parabolic equations [39], power functions [40], and exponential functions [41] were employed to depict the freeze–thaw deterioration of concrete. In general, the theories available to model freeze–thaw deterioration mainly include reliability theory, damage theory, and Grey theory. These theories can better describe the damage degree of concrete subjected to freeze–thaw cycles, which provides insights for the development of a damage model of ARSCC under freeze–thaw cycles.

Therefore, freeze–thaw cycle experiments on ARSCC were conducted in this study. The effects of AS and RCA on the frost durability of ARSCC were analyzed. Based on the freeze–thaw cycle experiment results, the freeze–thaw damage laws of ARSCC were investigated from both macro- and microperspectives. In addition, the response surface method demonstrated that the P_n_ better reflected the freeze–thaw degree of ARSCC damage than W_n_ and that it was more suitable to be used as the freeze–thaw damage variable of ARSCC. Furthermore, a freeze–thaw damage model for ARSCC was established based on the Weibull probability and Grey theories, which is of great significance for future research on and application of ARSCC.

There is a scarcity of studies in the literature documenting the incorporation of alkali silicates (AS) into recycled concrete. This study examines the frost resistance of recycled self-compacting concrete (ARSCC) when augmented with AS. The objective is to maximize the utilization of construction solid waste and alternative materials in concrete, thereby decreasing reliance on natural sand and gravel. This study aims to serve as a resource for the implementation of alkali-activated recycled construction waste concrete (ARSCC) in environmentally friendly concrete applications.

This paper solely focuses on the examination of the cold resistance of ARSCC. In order to effectively promote the advanced research on salt corrosion and concrete (ARSCC), it is recommended that future research endeavors focus on simulating real-world service environments. Specifically, the durability of ARSCC should be investigated under the combined influence of many factors, including salt corrosion, freezing and thawing, and loading.

## 2. Materials and Methods

### 2.1. Materials

**Figure 1:**
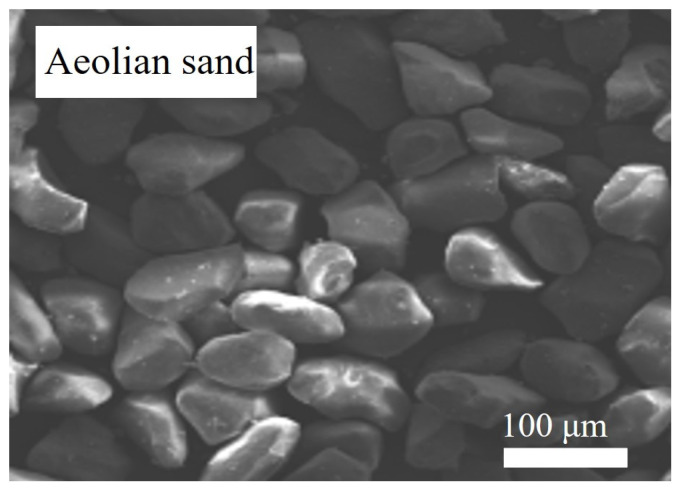
Aeolian sand microscopic morphology.

**Figure 2:**
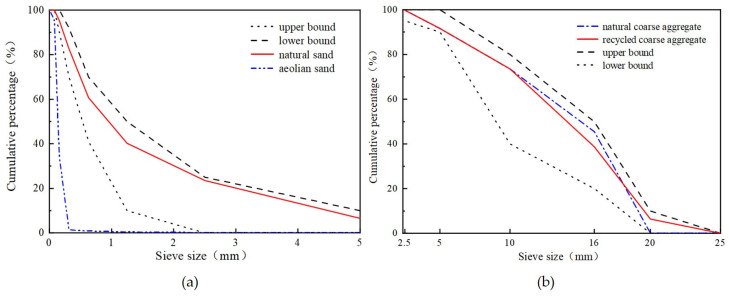
Sieve analyses of aggregate. (**a**) Sieve analyses of fine aggregate. (**b**) Sieve analyses of coarse aggregate.

Xinjiang Tianshan P. O 42.5 cement was utilized for the experiments. The Class II fly ash produced by the western construction of Urumqi was adopted. The water-reducing agent utilized was the polycarboxylic acid type, with a water-reducing efficiency of 25% to 30%. The mixing water was taken from the locally available domestic water in Urumqi. Natural sand (NS) was obtained from washed natural sand in the Xinjiang region with a fineness of 2.97 and an apparent density of 2487 kg/m^3^. AS was obtained from the Gurbantunggut desert in northern Xinjiang with a fineness of 1.25 and an apparent density of 2672 kg/m^3^. Its microscopic morphology is shown in Figure 1. Figure 2a shows the sieve analysis of the fine aggregate. It can be seen that the particle sizes in the AS were primarily centered at 100 μm. The AS predominantly exhibited an angular polyhedral morphology, with spherical particles of lower abundance and particle surfaces that appeared to be uniformly smooth. Table 1 presents their primary constituents. RCA was screened from the demolished concrete wastes in Xinjiang and processed by the mechanical crushing method. The apparent density and water absorption of the RCA were 2515 kg/m^3^ and 6.85%, respectively, while those of the NCA were 2700 kg/m^3^ and 0.52%, respectively. The grading curves for both are shown in Figure 2b. It can be seen that the gradings of RCA and NCA are similar and that both satisfy the requirements.

### 2.2. Mixture Proportions

**Figure 3:**
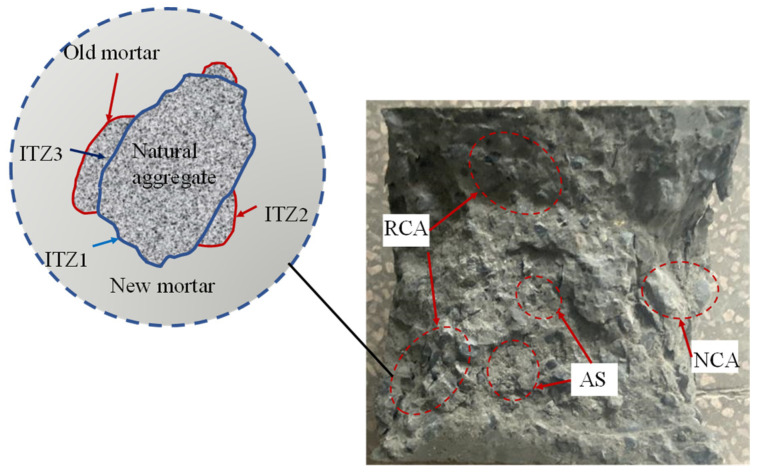
ARSCC internal aggregate distribution.

In this study, AS substitution rates of 0, 20%, 40%, and 60% as well as RCA substitution rates of 0, 25%, and 50% were designed for the experiments. Table 2 illustrates the composition of the ARSCC mixture. The distribution of the aggregates within the ARSCC is displayed in Figure 3. In the ARSCC, the interface structures of the aggregate–new mortar, mortar–new mortar, and aggregate–old mortar transition zones (ITZ1, ITZ2, and ITZ3) were particularly complex. AS could fill the pores between the coarse aggregate and cement paste.

### 2.3. Experimental Procedure

**Figure 4:**
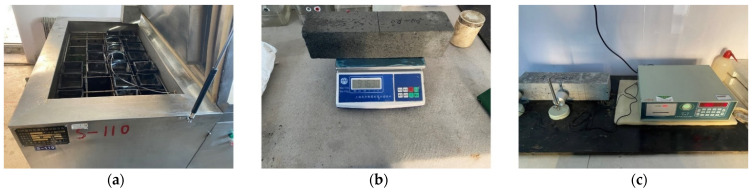
Specimen measurement: (**a**) freeze–thaw test equipment; (**b**) weight measurement; (**c**) transverse fundamental frequency measurement.

**Figure 5:**
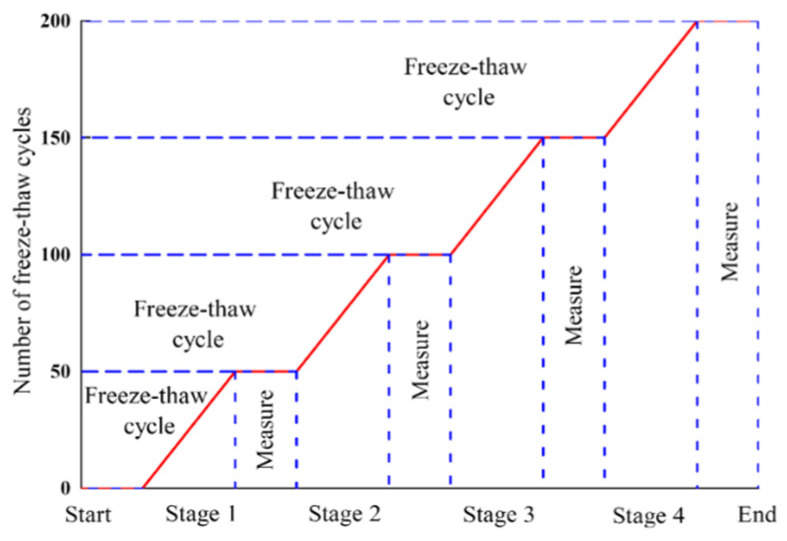
Schematic diagram of freeze–thaw cycle system.

**Figure 6:**
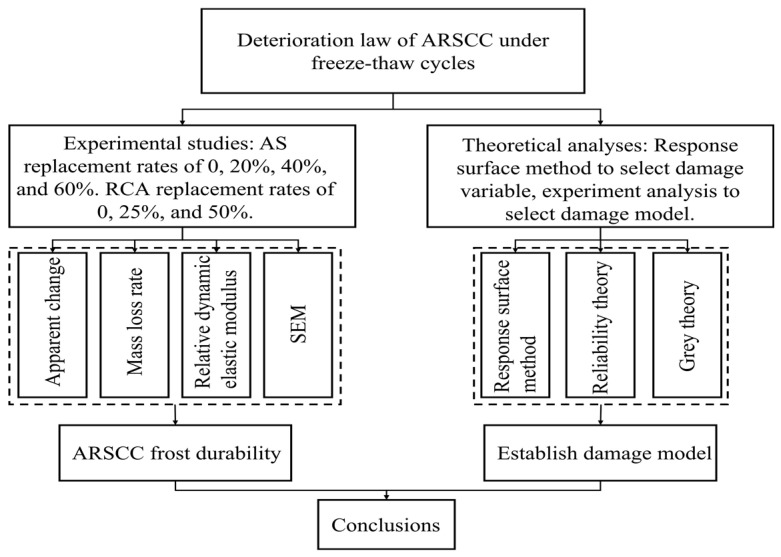
Schematic diagram of research route.

According to GB/T50082-2009 [42], the ARSCC specimens of 100 mm × 100 mm × 400 mm were prepared to determine the P_n_ and the W_n_ before and after the freeze–thaw cycle. The experiment employed in this study was the rapid freeze–thaw method with specific specifications [42]. The experiment apparatus was a TDR-type concrete rapid freeze–thaw tester (Figure 4a). The duration of each freeze–thaw cycle experiment was 4 h. For freeze–thaw cycles, freezing and thawing were performed at −18 ± 2 °C and 5 ± 2 °C, respectively. The specimens were removed every 50 cycles. The fundamental transverse frequency and saturated surface dry mass were measured using a DT-12 dynamic elastometer and a high-precision electronic scale (Figure 4b,c) (Shanghai Yousheng Weighing Apparatus Co., Ltd., Shanghai, China). Finally, the P_n_ and the W_n_ of the specimens were obtained. The specimens were considered damaged when P_n_ < 60% or W_n_ > 5%. Figure 5 indicates the freeze–thaw cycle regimes. Figure 6 illustrates the research route employed in the present study.

## 3. Results and Discussion

### 3.1. Changes in Appearance

**Figure 7:**
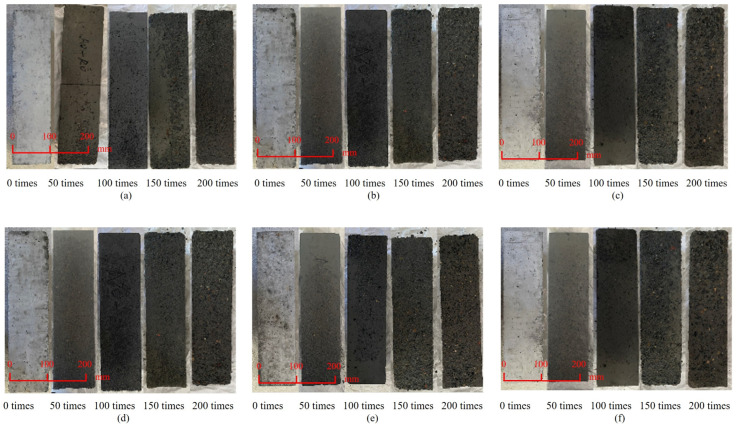
Apparent morphology of some ARSCC specimens after freeze–thaw cycles: (**a**) A0R0; (**b**) A20R0; (**c**) A20R50; (**d**) A40R0; (**e**) A40R25; (**f**) A60R50. Note: A—aeolian sand, R—recycled coarse aggregate; the subsequent numbers are substitution rates (%).

Freeze–thaw damages of the ARSCC specimens are depicted in Figure 7, illustrating their progressive deterioration over time. After 50 freeze–thaw cycles, the A0R0 and A20R50 specimens exhibited the weakest surface damages, as shown in Figure 7a,c. The damages were limited to sanding and minor mortar spalling on the specimen surfaces. After 200 freeze–thaw cycles, the spalling of the mortar on the surfaces of the A0R0 and A20R50 specimens increased. The apparent damage was similar for both. Figure 7b,d demonstrated that the surfaces of the A20R0 and A40R0 specimens were sanded after 150 freeze–thaw cycles. Figure 7e presented that the addition of RCA suppressed the AS-induced surface sanding. As shown in Figure 7f, the A60R50 specimen experienced the highest surface damage with significant mortar spalling at the corners. Table 3 shows the freeze–thaw cycle experiment results.

### 3.2. Mass Loss Rate

**Figure 8:**
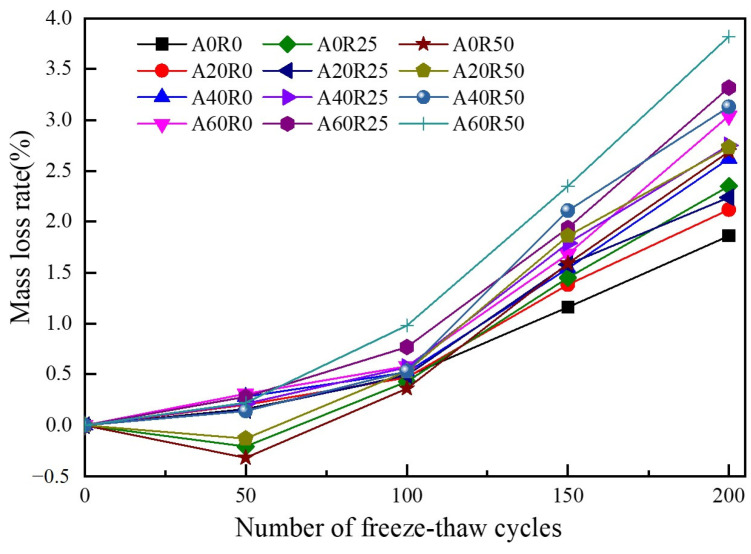
Variation of mass loss rate of concrete.

Figure 8 shows the variations in the W_n_ of the ARSCC specimens during the freeze–thaw cycles. The curves had a similar development pattern, with an initial slow and then a steep upward trend. The curve of the A60R50 specimen was the fastest growing one, with an average W_n_ of 1.84% for the four freeze–thaw stages and an average increase rate of 0.037% per stage. The A0R0 curve was moderate, with an average W_n_ of 0.91% for the four freeze–thaw stages and an average increase rate of 0.018% per stage. As shown in Figure 7, the maximum W_n_ of the concrete without RCA substitution was 0.31% (i.e., the A60R0 specimen), and the average of the four groups was less than 0.24%. When the RCA substitution rates were 25% and 50% and the substitution rate of AS was low, the specimens experienced negative W_n_ values during the initial 50 freeze–thaw cycles. This phenomenon can be attributed to the large porosity and high water absorption rate of the RCA [43]. Moreover, the concrete remained in an unsaturated water state until the freeze–thaw period. During the freeze–thaw cycles, the mass of the absorbed water increased faster than the loss mass to the surface mortar shedding due to water infiltration into the concrete through capillary absorption, thus exhibiting a negative growth in W_n_ on a macroscale. After 50 freeze–thaw cycles, the W_n_ of the ARSCC specimens gradually increased with the increase in the AS substitution rate. This could be attributed to the fact that, on the one hand, with the increase in the AS substitution rate, the appropriate addition of AS formed a continuous gradation with river sand [44], which effectively filled the significant detrimental pores in the RCA and decreased its water absorption. On the other hand, the increased deterioration of the surface mortar counteracted the ability of the RCA to absorb water and compensate for loss, which ultimately led to a decrease in the mass of the specimens. 

### 3.3. Relative Dynamic Elastic Modulus

**Figure 9:**
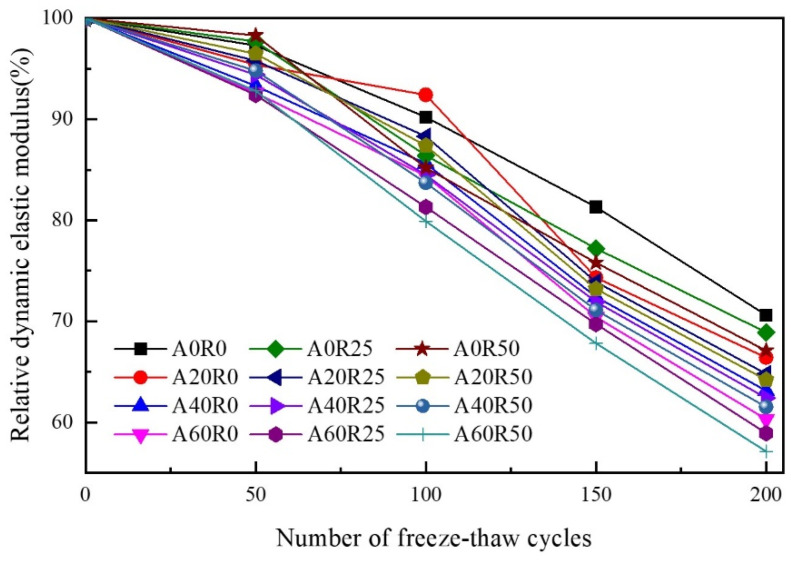
Variation of relative dynamic elastic modulus of concrete.

The effect of the freeze–thaw cycles on the P_n_ of the ARSCC specimens was confirmed by Figure 9, which exhibited first a slow and then a sharp downward trend. For the first 50 freeze–thaw cycles of the specimens containing a certain amount of AS, there were slight differences in the P_n_ values between the concrete groups. As the freeze–thaw process advanced, damage on the concrete surface mainly occurred in the early stage of the freeze–thaw cycles [45]. When the concrete was freeze–thawed for 100 cycles, it reached a rapid damage stage, resulting in a significant decrease in its P_n_. The A60R50 curve decreased the fastest, while the A0R0 curve decreased the slowest. The rate of decrease changed significantly when the AS substitution rate exceeded 40%. Throughout the freeze–thaw cycle, the P_n_ decreased with the increasing substitution rate of the AS for the ARSCC specimens without RCA addition. For the ARSCC specimens with RCA addition, the P_n_ increased and then decreased with the increasing AS substitution rate. 

Generally, the P_n_ can reflect the internal compactness of concrete from the side. During the freezing process, water freezing caused expansion pressure inside the concrete and the internal pores extended, which damaged the internal structure and reduced the P_n_ of the specimens. At the beginning of the freeze–thaw cycle, the stress generated inside the concrete was relatively small and the damage was mainly concentrated on the surface, and the P_n_ of each group of specimens changed gently. However, the freezing force increased as the number of freeze–thaw cycles increased, leading to an increase in the number of pores, which macroscopically manifested as a rapid decrease in the P_n_ of the concrete. Furthermore, with the increase in the RCA substitution rate, on the one hand, the structure of the weaker interfaces within the ARSCC specimen also increased, further enhancing the water transfer channels in the concrete. On the other hand, the increased width of the old aggregate and the new mortar reduced the resistance of the concrete to frost swelling force, resulting in increased internal porosity, higher water absorption, and reduced frost resistance. Appropriate addition of the AS could fill the large harmful pores of concrete, thus reducing the adverse effects of RCA. 

### 3.4. Bubble Spacing Factor

**Figure 10:**
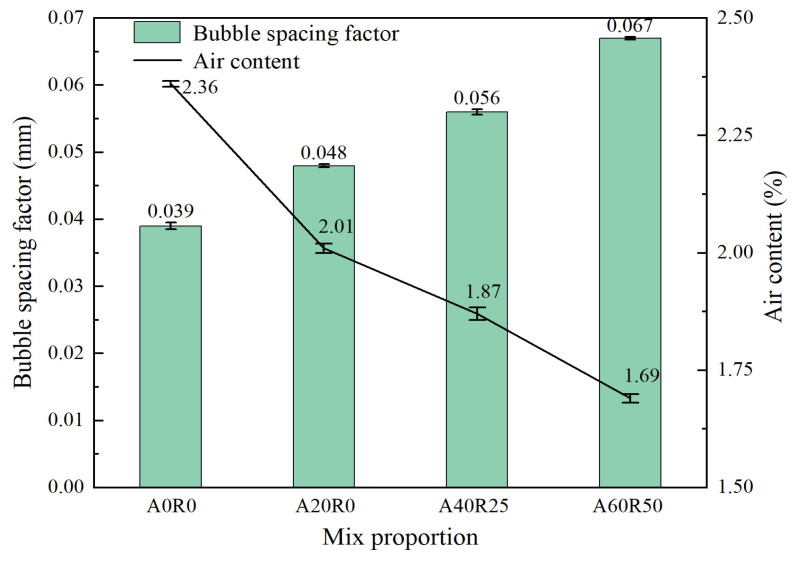
Bubble characteristic parameters.

The bubble spacing tester was used to calculate the bubble spacing factors of the ARSCC specimens. The smaller the bubble spacing factor, the smaller and more uniformly distributed the pores in the concrete and hence the better its frost resistance. Meanwhile, the air content was also related to the bubble spacing factor. A higher air content resulted in more bubbles in the concrete, thereby reducing the bubble spacing factor, which could be reflected in Figure 10. As can be seen in Figure 10, the A0R0 specimen had the smallest bubble spacing factor of 0.039 mm and the largest gas content of 2.36%. This indicated that the A0R0 specimen had better frost resistance. By increasing the contents of the AS and RCA, the bubble spacing factor increased while the air content decreased, thereby decreasing concrete durability. This was in accordance with the effect law of the AS and RCA on concrete in Figure 9.

### 3.5. Analysis of SEM

**Figure 11:**
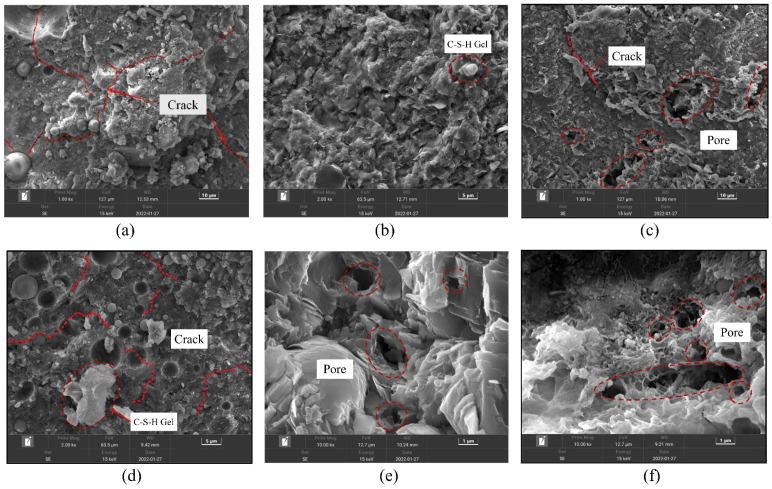
SEM morphologies of some ARSCC specimens: (**a**) A0R0; (**b**) A20R0; (**c**) A0R50; (**d**) A20R50; (**e**) A40R0; (**f**) A40R50.

**Figure 12:**
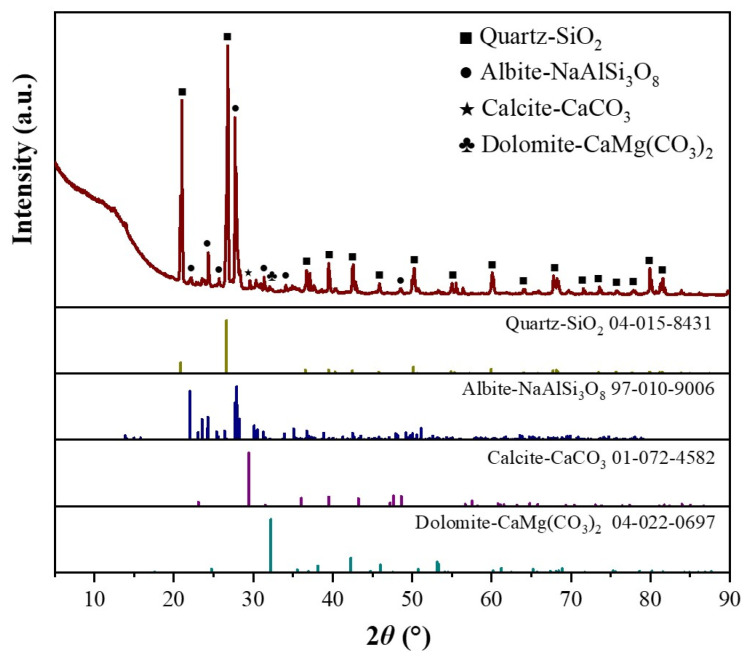
XRD of aeolian sand.

The macroscopic behavior of a material depends on its internal structure. Figure 11 shows the SEM morphology images of some ARSCC specimens. Figure 11a illustrates the initial cracks in ordinary concrete. As indicated in Figure 11b,e, the incorporation of AS filled some of the cracks in the concrete. Figure 11c confirmed that addition of the RCA increased the number of internal pores in the concrete. Figure 11d demonstrated that the addition of 20% AS and 50% RCA reduced the number of internal pores and increased the interfacial transition zone of the ARSCC. This indicated that AS could fill the cracks and pores of the concrete and compensate for the initial defect of large porosity of the RCA. Figure 11f revealed that the hydration products had diverse and complex compositions, with agglomerated calcium silicate hydrate (C–S–H) particles. As can be seen from Figure 12, AS is mainly composed of four minerals: quartz, calcite, albite, and dolomite. Combined with Table 1, it can be seen that the chemical composition of AS is mainly composed of SiO_2_, accounting for 75.09%. This indicates that AS can contain amorphous SiO_2_, which makes the AS have the volcanic ash effect [46,47]. The active components of the AS underwent volcanic ash reaction to produce C–S–H gel, which filled harmful pores and improved the internal structure of the concrete. According to Figure 10, when the AS substitution rate increased, the porosity decreased, which meant that the unfavorable effect of the RCA was weakened. In addition, the porosity also increased as the number of freeze–thaw cycles increased. Meanwhile, the tendency of increasing porosity gradually diminished as the AS substitution rate increased. This explained the trend of first the slow and then the sharp decrease in P_n_ in Figure 9.

### 3.6. Freeze–Thaw Damage Model

#### 3.6.1. Damage Variable Selection

**Figure 13:**
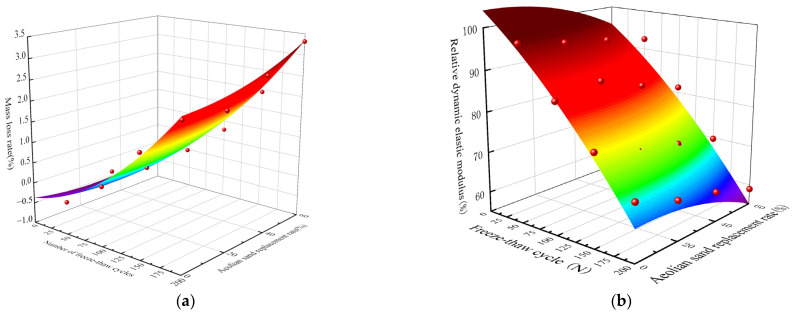
Response surface analysis: (**a**) mass loss rate response surface; (**b**) relative dynamic elastic modulus response surface.

Correlations between different responses and dependent variables can be established using response surface methods [48,49]. The freeze–thaw experiment results (Table 3) were utilized to ascertain the response surface of the W_n_ and the P_n_ using the Response Surface-Optimal module in the Design-Expert 13 software, which is illustrated in Figure 13. The response surface regression formula is given by Equation (1). The experimental and predicted values in Figure 13 exhibited a good agreement. The gradient of the response surface for the W_n_ was smooth (0.00005), while that for the P_n_ was steep (−0.0003) (see Figure 13a,b, respectively). These variations suggested that the number of freeze–thaw cycles and the AS substitution rate did not significantly affect the mass loss; however, they significantly influenced the P_n_. Due to the high porosity of RCA and the high water absorption of the AS, the W_n_ could not accurately reflect the damage caused by the freeze–thaw cycle. Furthermore, negative mass growth was observed in the early freeze–thaw cycles. These findings further demonstrated that, in contrast to the W_n_, the P_n_ provided a more accurate representation of concrete damage.

(1) $$\left\{ \begin{array}{c} W_{n}(N)=0.00005N^{2}+0.00022n^{2}+0.00007N\times n+0.004N-0.012n+0.0387\\ P_{n}(N)=-0.0003N^{2}-0.0027n^{2}+0.00006N\times n-0.143N+0.021n+111.624 \end{array}\right.$$

where *N* represents the number of freeze–thaw cycles, *n* denotes the substitution rate of AS, and *W_n_*(*N*) and *P_n_*(*N*) indicate the *W_n_* and *P_n_* for *N* freeze–thaw cycles and *n* AS substitution rates, respectively. 

Hence, the *P_n_* was used as the damage variable. According to damage mechanics theory, the degree of damage to concrete caused by freeze–thaw cycles can be described as:

(2) $$D=1-E_{n}/E_{0}$$

where *D* is the degree of concrete damage and *E_n_* and *E*_0_ represent the residual and initial dynamic moduli of elasticity of concrete, respectively.

#### 3.6.2. Establishment of the Freeze–Thaw Damage Model

Since the freeze–thaw damage of concrete is random, it can be analyzed by reliability theory. The Weibull distribution provides the theoretical basis for reliability and failure analysis and is widely used in reliability engineering [50]. Hence, the Weibull distribution model serves as a suitable solution for describing the freeze–thaw damage. The Weibull distribution model is categorized into two-parameter and three-parameter forms. The Weibull distribution model with three parameters, in contrast to the two-parameter Weibull distribution model, includes the location parameter *γ* that takes into account the initial freezing resistance of ARSCC. This coincides with the actual service life characteristics of concrete. In addition, to avoid the effects due to the correlation between parameters, the parameters of the Weibull distribution model can be determined using the Grey theory. The iterative process is avoided, and the fitting accuracy is high, fast, and accurate. Therefore, the three-parameter Weibull distribution model based on the GM(1,1) model was adopted in this study to model the freeze–thaw damage of ARSCC. The functional expression of the three-parameter Weibull distribution model is given below:

(3) $$F\left(t\right)=\{\begin{array}{cc} 1-\exp\left[-{\left(\frac{t-\gamma }{\eta }\right)}^{\beta }\right] & ,t\geq 0\\ 0 & ,t<0 \end{array}$$

where *t* is the number of freeze–thaw cycles, *β* is the shape parameter, *η* is the scaling factor, and *γ* is the position parameter.

A simple conversion of Equation (3) yields:

(4) $$t=\gamma +\eta \exp\left(\frac{\ln\left[-\ln\left(1-F\left(t\right)\right)\right]}{\beta }\right)$$

Let $t_{i}=\ln\left[-\ln\left(1-F\left(t_{i}\right)\right)\right],a=-1/\beta ,b=\gamma ,c=\eta$. Then, Equation (4) can be transformed into:

(5) $$t_{i}=ce^{-at}+b$$

**Figure 14:**
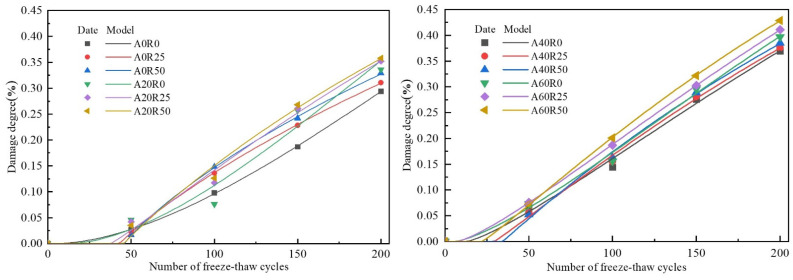
The three-parameter Weibull distribution model based on the GM(1,1) model.

Equation (5) has the same form as the solution of the Grey prediction GM(1,1) model. Hence, the parameters *a*, *b*, and *c* can be estimated using the GM(1,1) model, from which the parameters *β*, *η*, and *γ* of the three-parameter Weibull distribution model could be determined (Table 4). The obtained parameters were then utilized to construct the GM(1,1) model-based three-parameter Weibull distribution model. Figure 14 shows the constructed model.

As can be seen in Figure 14 and Table 4, the correlation coefficients (R^2^) of the fitted functions were high. The experimental values were in excellent agreement with the predicted values of the constructed freeze–thaw damage model. The results indicated that the model was able to accurately characterize the freeze–thaw damage rule of ARSCC specimens with varying substitution rates of the AS and RCA. When the substitution rate of RCA was zero, the shape parameter *β* approximately decreased with the increasing substitution rate of the AS. When the substitution rate of RCA was 25% or 50%, the shape parameter *β* rose as the AS substitution rate increased and then fell. These trends indicated that the frost resistance of the ARSCC without the RCA decreased with the increasing substitution rate of the AS. For the ARSCC specimens with RCA, the frost resistance increased and then decreased as the substitution rate of the AS increased. The scaling factor *η* and the location parameter *γ* decreased significantly with the increasing substitution rate of the AS. These trends indicated that the freeze–thaw damage rate of concrete accelerated with the increasing AS substitution rate. However, the scaling factor *η* and location parameter *γ* of the ARSCC specimens with 20% and 40% substitution rates of the AS were not significantly different from those of the ARSCC specimens without AS addition at each RCA substitution rate. Therefore, the optimum substitution rates of the AS and the RCA were 20–40% and 25–50%, respectively.

## 4. Conclusions

In this study, the damage deterioration laws of ARSCC under freeze–thaw cycles were investigated from macroscopic and microscopic perspectives. Then, the damage variables were determined using the response surface method. Based on the Weibull probability and Grey theories, the freeze–thaw damage model for ARSCC was established. The main conclusions can be summarized as follows:

1. The P_n_ of ARSCC gradually decreased as the number of freeze–thaw cycles increased. After 200 freeze–thaw cycles, the P_n_ values of the A20R0, A40R0, and A60R0 specimens decreased by 4.2%, 7.5%, and 10.3%, respectively, compared to the A0R0 specimen, while those of the A20R50, A40R50, and A60R50 specimens decreased by 2.9%, 5.6%, and 10.0%, respectively, compared to the A0R50 specimen. For the ARSCC without RCA, this decreasing trend gradually accelerated as the AS substitution rate increased. For the ARSCC specimens with RCA, this decreasing trend gradually slowed down and then accelerated as the AS substitution rate increased.
2. The laws of freeze–thaw damage gained from macroscopic and microscopic perspectives were consistent: appropriate addition of AS could reduce the unfavorable effects of RCA, thereby enhancing the frost resistance of ARSCC. Therefore, the optimum substitution rates of the AS and the RCA were determined to be 20–40% and 25–50%, respectively.
3. Based on the bubble spacing factor and SEM analysis, the P_n_ could describe the internal structure evolution of ARSCC. The accuracy of the P_n_ as a variable for the freeze–thaw damage of ARSCC was confirmed using the response surface method.
4. In combination with the results of freeze–thaw cycle tests as well as the Weibull probability and Grey theories, a freeze–thaw damage model that could accurately describe the freeze–thaw deterioration law of ARSCC was established.

In order to promote the eco-friendly and sustainable concrete studied in this paper vigorously in the future, aeolian sand and recycled coarse aggregate from different regions and countries should be taken for comparative analysis. Then, combined with the actual service environment, durability research under the multi-factor coupling of load–frost–thaw, salt corrosion–frost–thaw, etc., should be conducted. This study provides a reference for future practical applications.

## Figures and Tables

**Table 1:** Main components of fine aggregates.

Type	Chemical Composition of Aeolian Sand (%)							
	SiO_2_	Al_2_O_3_	CaO	Fe_2_O_3_	Na_2_O	K_2_O	MgO	Others
Natural sand	90.76	4.59	0.11	0.73	0.39	2.16	0.18	1.08
Aeolian sand	75.09	11.16	3.66	3.13	2.68	2.34	0.96	0.98

**Table 2:** ARSCC mixture compositions.

Type	W/B(%)	Amount of Material per Unit Volume (kg/m^3^)								
		C	F	W	AW	NS	AS	NCA	RCA	WS
A0R0	0.329	257.71	259.37	169.95	0.00	706.86	0.00	848.00	0.00	3.30
A20R0	0.329	257.71	259.37	169.95	0.00	565.49	140.8	848.00	0.00	3.30
A40R0	0.329	257.71	259.37	169.95	0.00	424.12	281.7	848.00	0.00	3.30
A60R0	0.329	257.71	259.37	169.95	0.00	282.74	422.6	848.00	0.00	3.30
A0R25	0.329	257.71	259.37	169.95	13.89	706.86	0.00	636.00	199.94	3.30
A20R25	0.329	257.71	259.37	169.95	13.89	565.49	140.8	636.00	199.94	3.30
A40R25	0.329	257.71	259.37	169.95	13.89	424.12	281.7	636.00	199.94	3.30
A60R25	0.329	257.71	259.37	169.95	13.89	282.74	422.6	636.00	199.94	3.30
A0R50	0.329	257.71	259.37	169.95	27.77	706.86	0.00	424.00	399.89	3.30
A20R50	0.329	257.71	259.37	169.95	27.77	565.49	140.8	424.00	399.89	3.30
A40R50	0.329	257.71	259.37	169.95	27.77	424.12	281.7	424.00	399.89	3.30
A60R50	0.329	257.71	259.37	169.95	27.77	282.74	422.6	424.00	399.89	3.30

Note: W/B— water to binder (cement + fly ash) ratio; A—aeolian sand; R—recycled coarse aggregate; the subsequent numbers are substitution rates (%); C—cement; F—fly ash; W—water; AW—additional water; NS—natural sand; AS—aeolian sand; NCA—natural aggregate concrete; RCA—recycled coarse aggregate; WS—water-reducing agent.

**Table 3:** ARSCC freeze–thaw test results.

Type	Relative Dynamic Elastic Modulus P_n_/(%)/Mass Loss Rate W_n_/(%)			
	50 Times	100 Times	150 Times	200 Times
A0R0	97.3/0.16	90.2/0.48	81.3/1.16	70.6/1.86
A20R0	95.4/0.2	92.4/0.47	74.3/1.38	66.4/2.12
A40R0	93.3/0.28	85.6/0.52	72.4/1.54	63.1/2.62
A60R0	92.6/0.31	84.4/0.58	70.4/1.68	60.3/3.04
A0R25	97.7/−0.21	86.4/0.43	77.2/1.45	68.9/2.35
A20R25	95.8/0.15	88.3/0.49	73.9/1.58	64.8/2.24
A40R25	94.4/0.21	84.5/0.57	71.9/1.79	62.4/2.75
A60R25	92.4/0.28	81.3/0.77	69.7/1.94	58.9/3.32
A0R50	98.3/−0.32	85.2/0.36	75.8/1.59	67.1/2.69
A20R50	96.5/−0.13	87.3/0.53	73.2/1.86	64.2/2.73
A40R50	94.8/0.14	83.7/0.53	71.1/2.11	61.5/3.13
A60R50	92.8/0.22	79.9/0.98	67.8/2.35	57.1/3.82

**Table 4:** Parameters of the three-parameter Weibull distribution model based on the GM(1,1) model.

Type	*β*	*η*	*γ*	R^2^
A0R0	1.7092	360.7104	5.6158	1.0000
A20R0	1.6878	305.6317	13.2728	0.9748
A40R0	1.2650	344.2264	12.9673	0.9933
A60R0	1.3586	319.3656	5.9991	0.9944
A0R25	0.9088	467.3117	43.2388	1.0000
A20R25	1.0914	348.3152	37.6027	0.9829
A40R25	1.0720	345.0348	29.1663	0.9968
A60R25	1.2709	318.4103	7.1753	1.0000
A0R50	0.8715	444.6070	46.1441	1.0000
A20R50	1.6131	363.2361	45.6812	0.9814
A40R50	1.0044	340.6668	34.4007	0.9976
A60R50	1.0973	302.2938	22.0165	1.0000

## Data Availability

Not applicable.
